# Adolescents in a tuberculosis hospital: Qualitative study of how relationships with doctors, caregivers, and peers mediate their mental wellbeing

**DOI:** 10.1371/journal.pone.0257379

**Published:** 2021-10-01

**Authors:** Olga Zvonareva, Saskia Witte, Nadezhda Kabanets, Olga Filinyuk

**Affiliations:** 1 Department of Health, Ethics and Society, Maastricht University, Maastricht, The Netherlands; 2 StrAU Integrative Approaches to Public Health and Health Care, Siberian State Medical University, Tomsk, The Russian Federation; 3 Policy Analysis and Studies of Technologies Center, National Research Tomsk State University, Tomsk, The Russian Federation; 4 Department of Tuberculosis and Pulmonology, Siberian State Medical University, Tomsk, The Russian Federation; The University of Georgia, UNITED STATES

## Abstract

Lengthy hospitalization can impact adolescents’ mental wellbeing in a number of negative ways. Scholarship has indicated that a young patient’s relationships play an important role in reducing the amount of stress felt and in improving emotional state. In this article we turn to the experiences of adolescents with tuberculosis [TB] in Russia to explore how exactly hospitalization together with the TB diagnosis itself impact their mental wellbeing and how relationships with others mediate these impacts. We conducted a qualitative, interview-based study in Tomsk pediatric TB clinic. Interviews were conducted with three groups relevant for reaching the aim of this research: adolescent patients, their adult caregivers, and their treating physicians [17 informants in total]. Interview data were complemented with prolonged observations in the same clinic. The results of our study highlight that threats to mental wellbeing of adolescents with TB are multiple. Adolescents who are about to enter the in-patient treatment feel apprehensive and anxious about their future. They tend to have a hard time accepting their diagnosis, which they often feel is something shameful, and, consequently, may develop a negative attitude towards themselves. Most importantly, many undergo painful loss of personal relationships and expect or actually experience rejection by peers because of having tuberculosis. However, relationships with physicians, caregivers, and other patients in the clinic mediate negative impacts of TB diagnosis and hospitalization on adolescents’ mental wellbeing and can open ways for providing support. Supportive practices include physicians leaving it up to adolescents to decide what they want to discuss and when, caregivers remaining available for contact and keeping regular communication, and other adolescents with TB proactively seeking contact with the newcomers and behaving in a non-judgmental way. These results can inform design of adolescent-friendly TB services.

## Introduction

Hospitalization is a stressful event for young patients [1]. They arrive to an unfamiliar environment and are separated from relatives and friends. Often, they experience negative emotions, such as anxiety and fear [2, 3]. For adolescents in particular among the main problems in a hospital ward is loss of independence and autonomy to which they may react with anger, frustration, or social withdrawal [4, 5]. At the same time, studies indicate that a young patient’s relationships with relatives and healthcare workers play an important role in reducing the amount of stress felt and in improving the state of mental wellbeing [6–8]. The positive interactions with relatives and health care workers can aid in the adolescent’s coping process through the provision of both social as well as emotional support and disease- and therapy-related information. In this article we turn to the experiences of adolescents with tuberculosis [TB] in Russia for an in-depth exploration of how prolonged hospitalization together with the TB diagnosis itself impact their mental wellbeing and how relationships with others mediate these impacts.

TB in adolescents has largely been overlooked in research and practice efforts to address the global burden of this disease. The childhood tuberculosis epidemic has been recently drawn into the spotlight after decades of neglect [9]. Yet adolescence, spanning the ages of 10–19, deserves special attention. Such attention is needed because, firstly, adolescence is associated with an increased risk of TB exposure and disease [10] and, secondly, adolescents are particularly vulnerable to poor treatment outcomes due to peer-pressure and fear of social isolation, specific psychosocial needs, and unique challenges related to autonomy and adherence [11, 12]. Importantly, TB is known to take a heavy toll on patients’ mental health [13, 14]. Adolescents, specifically, experience depression and may internalize TB-associated stigma which leads to poor self-esteem [15]. The recent Roadmap Towards Ending TB in Children and Adolescents stated that ‘10–19 year-olds need adolescent-friendly services that include relevant psychosocial support and minimal disruption of education’ [9, p.11]. However, development of such services is hindered by little knowledge available specific to this age group [16]. In this article we contribute towards filling this gap by focusing on the mental health needs of hospitalized adolescents and attempts to respond to these needs in a particular sociocultural context of Russian TB care.

We follow scholars who define mental wellbeing in terms of the degree to which a person is fully functioning or, in other words, realizing their human potential, including the potential to meaningfully relate to others [17, 18]. Psychology and related disciplines have increasingly appreciated the fundamental importance of warm, supportive, and trusting relationships for all aspects of mental wellbeing [19, 20]. Importantly, it is the quality of relatedness that is supportive of mental wellbeing, not just the quantity of interactions [21]. Scholarship suggests that experiences of greater relatedness are facilitated by, among others, feeling understood, engaging in meaningful dialogue or activities together, or having fun with others [22–24]. Not surprisingly, for adolescents, who this study focuses on, quality of relationships with parents is of primary importance and has short and long-term consequences for mental wellbeing [25]. Relying on the delineated understanding of mental wellbeing which emphasizes primacy of relationships, in this study we take relationships as an entry point into investigation of mental wellbeing of hospitalized adolescents with TB. How do relationships mediate impacts of the hospitalization and the diagnosis of TB itself on adolescents’ mental wellbeing? Can we capitalize on the role of relationships to support adolescents in treatment in the hospitals?

Discussion is ongoing within the TB research and policy community about the place of hospitalization in treatment of children and adolescents with TB. The prevailing opinion is that unnecessary hospitalization is to be avoided and services are to be reoriented towards decentralized community-based care [26, 27]. Many participants of this discussion are concerned with disruption of education and extracurricular activities experienced by hospitalized children and adolescents and argue that move away from routine hospitalization allows more patient-centered and cost-effective care. At the same time, when making a decision about hospitalization in practice, clinicians often weigh a number of considerations pertinent to a particular patient. These considerations include host-related conditions such as immune and nutritional status, TB-related clinical conditions such as disease severity and drug resistance, and social and logistic criteria such as living conditions, availability of TB expertise at the community care level, and availability of committed social/parental support for treatment [28]. In a nutshell, it is unlikely that one-size-fits-all model for TB treatment can be suitable for all children and adolescents. At least in some situations hospitalization will continue to be employed in the future to mitigate risks of severe disease and death. Therefore, studies such as the one reported in this article are important to provide insights on how hospitalization can be made more supportive of mental wellbeing of young patients with TB. These insights can also be relevant for other conditions that require hospitalization.

## Methods

Siberian State Medical University Research Ethics Committee approved this study. All informants signed informed consent form.

This is a qualitative, interview-based study. Interviews were conducted with informants from three groups relevant for reaching the aim of this research: adolescent patients, their adult caregivers, and their treating physicians. Involving these three groups allowed the research team to reach a grounded understanding of how the relationships are experienced and shaped. While standardized measures for different kinds of mental wellbeing exist, such as Psychological Wellbeing Scale [29] and Social Wellbeing Scale [30], we turned to informants’ narratives about tuberculosis, hospitalization, and relationships told in their own words. Such qualitative approach opened a window into interpretations, reasoning, and concerns involved into making sense of TB diagnosis and time in the hospital. We, consequently, were able to discern how and why hospitalized adolescents with TB experience changes in their mental wellbeing and to identify moments where support can be provided.

### Setting

The World Health Organization [WHO] classifies the Russian Federation as a high TB burden country. WHO estimated that in 2018 the TB incidence rate there was 54 per 100 000 people [31]. Of the total incidence rate reported in 2018 it is known that 3% were individuals younger than 15 years old [31].

This study was conducted in the Russian city of Tomsk with a population of about half a million. Tomsk is the administrative center of Tomsk Oblast, a region located in the Western Siberia. Throughout Russia, TB care for individuals younger than 18 is typically organized on a regional level, which means that children and adolescents with TB from the entire Tomsk Oblast usually have their diagnosis confirmed and receive treatment in Tomsk in a specialized pediatric TB clinic. Treatment trajectories of most patients in this clinic start from a routine yearly screening in an educational facility. Children and adolescents with test results concerning for TB are assessed at a specialized TB clinic and are invited for in-patient treatment if TB is confirmed.

The main guiding policy for TB treatment in Russia is the decree #932n by the Ministry of Health ‘On approval of the procedure for providing health care to people with tuberculosis’. While this document does not state anything specifically about hospitalization of children and adolescents with TB, the most recent clinical recommendations ‘Tuberculosis in children’ prepared by the Russian Society of Phtisiatrists and approved by Russian Ministry of Health in 2020 suggest 9 reasons for hospitalization of patients younger than 18 years old:

- Treatment of active TB of any localization during the intensive therapy phase
- Sputum smear-positive pulmonary TB
- Drug resistant TB [confirmed or suspected]
- Extensive, destructive and complicated forms of TB
- Life-threatening conditions [including blood spitting]
- Necessity of application of special methods, including surgical ones, for diagnosis and differential diagnosis of TB
- TB with comorbidities that require in-patient treatment and observation [HIV, diabetes, etc]
- Preparation for surgical treatment
- Combination of medical, epidemiological, and social indications for hospitalization.

In practice Russian TB specialists tend to be worried about lack of expertise outside specialized TB facilities to administer drugs in correct, age-friendly doses, to determine response to therapy, and to detect adverse events early. Another related source of concerns for them is the proximity of patients to a healthcare facility with pediatric TB experience since many of their young patients may live in settlements outside of regional centers such as Tomsk and may not be able to consult a TB specialist quickly in case the need arises, while local primary health care may not be able to provide adequate support. Finally, diverse ‘social indications for hospitalization’ mentioned in the clinical recommendations are important for pediatric TB specialists, who tend to be concerned whether a patient’s family is capable and willing to ensure conditions for lengthy treatment. As a result, in-patient treatment of children and adolescents with TB, at least during its initial phase, is almost always the default choice.

The clinic where this study was conducted is a state inpatient healthcare facility that provides TB treatment to minors who reside within the Tomsk Oblast. The TB clinic offers care to school-aged children and adolescents [7–18 years] and has in total forty-two beds. There is an isolation ward equipped to treat patients with resistant forms of TB and a general ward, where patients share space. The clinic staff consists of three TB specialists, two psychologists as well as nurses and tutors. Presence of tutors is meant to ensure that hospitalized patients continue their education away from their school.

### Informants

Fieldwork for this study took place in May-July 2018 in the Tomsk pediatric TB clinic. All patients in the age group of 12–18 years old who were treated in the clinic at the time of fieldwork were approached with an invitation to participate in the study. The adolescents receiving treatment in an isolation ward for drug resistant forms of TB were not approached. Almost all approached patients agreed to participate, which resulted in eight patient-informants, three of whom were boys and five were girls, aged 13–16 years. The duration of their in-patient treatment at the moment of study enrolment varied from 1 month and 9 days to 7 months and 6 days. Caregivers of the adolescent informants were invited for study participation as well. Per adolescent, one primary caregiver was approached. In total five mothers and one grandmother took part in interviews [Table 1]. Finally, all three physicians who treat school-aged patients in the TB clinic took part in the interviews. In total seventeen informants were involved.

**Table 1:** Adolescents and their caregivers.

Number	Age	Gender	Duration of stay in the TB clinic at the study onset	Caregiver interviewed
1	14	Male	3 months, 28 days	Mother
2	16	Male	5 months, 10 days	No interview [Large distance between the clinic and family home precluded caregiver’s visit to clinic as well as home-visit by researchers]
3	16	Female	3 months, 10 days	Grandmother [Custody over the child is with the grandmother. The grandmother is therefore the first caregiver of the adolescent patient]
4	15	Female	4 months, 0 days	Mother
5	15	Female	1 month, 9 days	Mother
6	15	Male	7 months, 26 days	Mother
7	13	Female	1 month, 27 days	No interview [Multiple appointments were scheduled, none were successful.]
8	15	Female	7 months, 6 days	Mother

### Data gathering

In this study data were generated, primarily, by conducting narrative interviews [32]. Narrative interviews focus on eliciting a story of an informant, instead of emphasizing a question-answer format [33]. Interview guides, therefore, involved broad questions aimed, first, at triggering the process of narration and, second, at following up on the provided narrative to ascertain details and reasoning [34]. The interviews lasted between forty-five minutes and an hour and a half and provided informants with space to tell their story of becoming sick and getting treatment [in the case of adolescents], of caring for a sick child and dealing with TB in the family [in the case of caregivers], and of treating patients and finding ways to deal with challenges involved [in the case of physicians]. Interviewer interfered minimally in the informants’ stories in order to preserve the logic, important details, and turning points as seen by the informants themselves. During interviews with informants from all groups particular attention was paid to their personal experiences of interacting with others and effects of these interactions. Interviews were arranged on the individual basis. The interviews with the adolescent patients, physicians, and some caregivers took place in a private room at the TB clinic. Interviews with other caregivers took place at the family homes in response to personal preferences of informants. All interviews were conducted by the same researcher [SW] in Russian language with translation between English and Russian provided by a senior student from the local medical university. Interviews were audio recorded with the permission of the informants and English language parts of the interviews were transcribed verbatim. The narrative interview data were further enriched and contextualized by observations made by SW with the assistance of translator of the verbal and non-verbal interactions between the adolescents, staff, and the visiting caregivers. Often present in the TB clinic for prolonged periods of time, she was also able to ask additional and clarifying questions during multiple non-structured conversations. These data were preserved in the form of field notes written on the same day as an observation or interaction took place.

The interviewer [SW] was trained in qualitative interviewing as well as the translator. Interview guides and process were developed jointly by the study team members with complementary expertise: qualitative health research [OZ]; TB treatment in Russia and impacts of TB on mental health [OF and NK], and global health [SW]. Additionally, external expertise on mental wellbeing was solicited from members of Department of Psychology and Behavioral Medicine in Siberian State Medical University. Nobody among the study team was involved in the care of the adolescent patients in the clinic where this study was conducted.

### Data analysis

In this study data were analyzed using thematic content analysis approach [35]. Analysis was informed by the theoretical concept of mental wellbeing and its emotional, psychological, and social components. Emotional wellbeing refers to presence of positive affect, relative absence of negative affect, as well as life satisfaction define mental wellbeing [36]. Psychological wellbeing covers more personal and private aspects of wellbeing and highlights that people are mentally well when they ‘like all parts of themselves, have warm, trusting relationships, see themselves developing into better people, have a direction in life, are able to shape their world to satisfy their needs, and have a degree of self-determination’ [20, p.300]. Social wellbeing covers more public and engaged aspects of wellbeing and highlights that people are mentally well when ‘they view social life as meaningful and understandable, when they see society as possessing potential for growth, when they feel they belong to their communities, are able to accept all parts of society, and when they see their lives as contributing to society’ [20, p.300]. As is typical for qualitative studies, data gathering and preliminary analysis proceeded simultaneously. To guard against overreliance on a particular analytical perspective, entire team discussed the emergent data, their significance, and meaning. Emerging insights from the field were regularly discussed by the entire research team to inform subsequent interviews and select points that required further clarification. Concurrently interview transcripts and field notes were read to identify recurrent themes [37], compare them across adolescent patients, caregivers, and physicians, and reflect on the meaning and significance of convergencies and divergencies. Collectivity of this process, including discussion of alternatives and identification and mitigation of the differences, guarded against uncritical acceptance of interpretations. With the arrival of more data, the identified themes acquired more precision and were used to generate codes. Thus, the final set of codes was, both, informed by the existing literature on mental wellbeing and derived from the generated data. After testing and refining, the codes were applied to the entire dataset for systematic analysis [38, 39], using ATLAS.ti.

### Ethical considerations

The participating adolescents as well as their caregivers and the treating physicians, were provided a participant information sheet about the study background, purposes, investigators, participants’ role, and how data will be used and stored. All were also asked to complete an informed consent form prior to study participation. Wording of information sheets and informed consent forms were tailored to the informant group. For the adolescents under the age of sixteen, a caregiver’s signature was also required for study enrolment. It was stressed, specifically for adolescents, that they were not required to participate and that their choice to accept or decline participation in the study would not have any consequences for their care. All informants also received contact details of Siberian State Medical University Research Ethics Committee, which reviewed this study.

## Results

In this section we analyze how relationships of the adolescent patients with physicians, caregivers, and peers, develop and are experienced. To illustrate the informants’ viewpoints quotes from their narratives are provided. To preserve the anonymity of the informants, they are identified with codes (see Table 2).

**Table 2:** Informant codes.

Element of the code	Meaning
P	Patient
C	Caregiver
D	Physician
1…8	Informant’s assigned number
M	Male
F	Female
12 … 16	Age [only for patient informants]

For example, a code #P1M14 refers to a male patient number one, fourteen years old.

### Relationships with physicians

Upon arrival to the TB clinic, patients and their caregivers are welcomed by a physician. The initial patient-caregiver-physician interaction takes place in a separate room. When the caregivers were asked during the interview to share their initial experiences at the clinic, they mostly described this introductory meeting as something when papers needed to be signed. For example, one caregiver responded when asked about the arrival to the TB clinic: ‘Well, I don’t remember a lot. We came here, filled some documents. This is it’ [#C1F].

Both patients and their caregivers also recalled with appreciation that physicians readily shared information about TB and its treatment during the introductory meeting. The introductory meeting in the TB clinic tends to rely on one-way communication with the physician taking on the speaker’s role and patients together with their caregivers mostly settling for listening. The latter report feeling content with such arrangement, feeling the need to know more about what is happening and showing relatively little interest in sharing their personal stories with the physician. This little interest may stem from a perception that the treating physician is already well-informed about the condition of newly arriving patients. As one informant who was about to be hospitalized for treatment stated: ‘Well, the doctor already knew everything’ [#P7F13] to explain why the physician did not ask her personal questions during the introductory meeting.

Physicians, on the other hand, are primarily concerned with thwarting potential refusals of hospitalization and attempts to cut the hospitalization period short. One question that physicians do receive regularly from the caregivers is why the treatment process cannot be sped up. Some caregivers try to open a discussion by expressing their dissatisfaction with the treatment duration. One of the interviewed physicians reported referring to the national guidelines and regulations when trying to convince caregivers that hospitalization is necessary, and treatment cannot be sped up:

We have a period of time for treatment and we follow federal clinical recommendations. I even always quote this recommendation for parents who are trying to hurry me up. And I say that if I try to make the treatment process faster, it will have a bad outcome. Sometimes they still ask to do it faster. I tell them; ‘no, we do it as it has to be done’.

[#D2F]

When anticipating caregivers’ refusals to let a patient be hospitalized, physicians try to address it first, before turning to any other topic. In such cases adolescents and their caregivers are approached in a very direct and authoritative manner as illustrated by experience of one mother:

They [medical staff] came out and explained that we had to get her hospitalized. Even if you don’t want to stay there, we will take her anyway and she will anyway get hospitalized. That we will have to get cured.

[#C4F]

It is after the potential refusals of hospitalization and attempts to shorten the treatment duration are fought off and adolescents are admitted to the clinic that physicians begin to actively focus not only on medical treatment itself but also on mental wellbeing of their patients. In contrast to the initial interactions during the introductory meeting, throughout the period of hospitalization physicians tend to leave it up to the adolescents to shape their relationships with medical personnel. This arrangement is appreciated by patients because it allows them to enjoy some autonomy in the otherwise quite constrained environment. Right away, a physician informs his or her patients that it is possible to discuss any topic at any time the adolescent feels the need. The opportunity to do so is facilitated by the ‘open door’ policy, shared by the physicians at the clinic. Open door policy entails that throughout the day, all adolescents are welcome to consult their treating physician on matters of their concern–an opportunity most patients use on a regular basis. Adolescents commonly expressed appreciation for an opportunity to speak to the physician at any moment a question arises:

When I am visiting a doctor, I ask her questions and she always answers me. For example I am asking about my last medical examination, if the result was good or not. Any time I ask, she will always answer me.

[#P6M15]

Often the patients’ visits are motivated by the need to know about the state of their health and the treatment progress. During the initial weeks in the TB clinic, adolescents are busy with adapting to the clinic routines. Then many of the newly admitted patients gain strong interest in dynamics of their own health condition. Physicians meanwhile patiently wait for adolescents to express the desire to have a conversation and also leave it up to a particular adolescent to decide on how much and what exactly he or she wants to know and how often the updates are to be provided. Many patients make it their priority to learn about TB and their treatment, as illustrated by one adolescent’s response to the question about what he finds most important in the relationship with his physician:

I want to be provided with the correct information. I want to know everything about my disease. I want to be provided will all information

[#P2M16]

Receiving timely answers to personally formulated questions makes adolescent patients feel more competent and confident with respect to the commonly shared goal–successful completion of the treatment. At the same time some patients show less interest in communicating with their physician and prefer to assume more passive role in the treatment process. Physicians respect this approach as well and don’t force unwanted interactions in such cases.

Apart from responding to what can be called cognitive needs of the patients, physicians attempt to respond to their emotional needs as well. Interviewed physicians believed in the beneficial effect of adolescents’ positive mood on the treatment process and for this reason tried to facilitate emotional wellbeing of their patients. Physicians indicated that, in order to provide emotional support, they try to invite adolescents to share both positive and negative emotions with them. While sometimes patients take the initiative in sharing their emotions with the receptive physicians, in other situations physicians feel obliged to offer their support more actively. For example, one physician narrated:

Or you can get some information here and now, for example you see that this kid is upset and you see his red eyes after crying and his lips are hanging. In that case you cannot just keep going and you will ask this child about what has happened.

[#D2F]

By asking the patient about the reasons for feeling sad, physicians actively invite sharing worries and demonstrates willingness to provide support in difficult moments. The likelihood of patients seeking emotional support from the physicians is further increased by the physicians’ attitude towards negative mood as something normal. The negative mood is typically depicted as something one can openly speak about in the TB clinic and for which one does not have to be ashamed.

The already quoted physician further elaborated that, in her opinion, in most cases an adolescent’s sadness is due to losing touch with their families. All three interviewed physicians admitted to regularly encounter situations when caregivers are unable to maintain regular communication with a patient by calling and/or visiting. Physicians report they feel the need to make up for such instances. The interview narratives show that the physicians do this by inventing reasons for why the caregiver could not contact the patient:

Sometimes parents don’t call them for a long time. They [patients] are worried and we [physicians] are trying to keep them calm and say that their parents are just busy, or they don’t have money or something else.

[#D3F]

Apart from such attempts to make up for irregularities in contact between caregivers and patients, all physicians also indicated they try to lower the number of occasions when a caregiver does not call or visit the patient. In the absence of other means to support the contact between caregivers and patients, interviewed physicians reported a tactic of calling a caregiver, after having calmed down a sad patient. The statement by one of the physicians shows the direct manner in which a caregiver is typically approached:

Then you call this mother and criticize her behavior. Even if you have something to do, you should leave it and come here, because your child is waiting for you.

[#D2F]

The same physician continues her story by telling that all of this is done in the absence of the patient: ‘We always try to protect the parents in the eyes of the children’. The reason is a belief that when the criticism of the physician towards the caregiver is expressed in the presence of the patient, this can negatively influence the adolescent’s perception of his or her relationship with the caregiver with further negative consequences for the adolescent’s mental wellbeing.

Overall, while not having much say in the treatment decisions, adolescent patients in the TB clinic have considerable autonomy over what to discuss with the treating physician and over the extent of the physician’s emotional involvement with the patient’s daily life in the clinic. Physicians interact with their patients in a flexible manner, responding to cognitive and emotional needs of each individual patient.

### Relationships with caregivers

Adolescents who receive treatment at the TB clinic come from a variety of family backgrounds. Physicians stress that while tuberculosis is popularly viewed as ‘disease of the poor’, in the TB clinic they see patients from all walks of life. At the same time, during the interviews all physicians conveyed that their patients who do come from unfavorable environments tend to experience much strain due to the circumstances of their daily life. It is not uncommon for adolescents with TB to lose a family member to the same disease, since minors are prone to contracting TB from family members with whom they live in close proximity for a long time. For example, one patient [#P3F16] came to the TB clinic from an orphanage where she had lived after her mother died from TB. Other problems sometimes faced by the patients treated in the TB clinic include substance abuse by the caregiver and little freedom at home due to a demanding list of chores in a financially constrained household.

Yet, despite the large array of possible difficulties, the majority of the patients interviewed described having a positive relationship with at least one adult caregiver. This adult tends to be female, while male caregivers such as fathers were seldomly mentioned during interviews. When patients mentioned their fathers, it was always in addition to their mother or grandmother, never individually. Illustrative here is a narrative by a teenage girl who lives with her father and grandmother since her mother died from TB. She first described how she sees her father:

My father is coming home. He is sitting and watching a movie. Well, sometimes when he is coming home, he is very tired, and he is just sitting and going to sleep.

Then she proceeded to describe her relationship with her grandmother:

She is communicating with me more than my father, because she is always at home and my father is always at work.

[#P7F13]

Here, it is a female household member who takes on the responsibility of care, whereas the task of breadwinning is put on the male household member. The described situation, where the grandmother functions as primary caregiver is also not unusual. In those instances when the mother passes away, the grandmothers appear to be the first to take on care for the child.

During the interviews, patients described their caregivers’ practices that made them feel supported and helped dealing with the long-term hospitalization. Initially, in the process of admission to the TB clinic, it proved to be of paramount importance that an adolescent is accompanied by at least one caregiver. Adolescents who were not alone during the initial appointment with the TB clinic physician shared similar accounts, such as ‘They [parents] stood by my side’ and ‘I was feeling not alone. I felt my mother will be close by in every situation’. These accounts indicate that presence of caregivers helps adolescent patients feel more secure in the face of unfamiliar situation. These first moments in the hospitalization trajectory are especially important since this is when a doctor communicates the exact diagnosis and treatment plans. Being together, patients and their caregivers can reflect jointly on what they are being told and what the diagnosis and hospitalization mean for them.

Many adolescents who arrive to TB clinic have never been hospitalized before and are uncertain about what to expect. Caregivers, therefore, can affect the initial attitude of the patient towards the TB clinic. An example of how a parent does this was provided by a woman who, after the diagnosis was communicated, immediately offered her son to focus on positive aspects:

After we passed the medical examination, I told him that something was wrong. And he said: ‘Oh, I will be in the hospital for two weeks?’. I had to tell him it would be more than two weeks. When I was telling him, the good thing is that sickness is not showing itself. Nothing hurts and he doesn’t feel anything bad. The treatment will happen close to home and I will be able to come to you every day if needed. That there won’t be any difficult surgeries, etc.

[#C9F]

Caregivers, thus, can try to help adolescent patients feel less anxious at the doorstep of the TB clinic by picturing the long-term hospitalization as something unusual, but manageable, detailing how hospitalization can be dealt with, and by affirming their support.

Interviewed adolescents also repeatedly referred to their caregivers’ confirmation for the need of hospitalization. Most stated that they were told by their caregivers in a direct manner that they had to undergo the treatment at the TB clinic, which helped to make the prospect of spending months away from home reasonable and acceptable for them. One such an example is provided by a girl, who said:

I have never been in such clinics for such a long time. But my grandmother told me that I have to, to get healthy and everything will go away, everything will be fine.

[#P3F16]

When the caregiver immediately expressed his or her approval of the physician’s decision for hospitalization, not much room for doubt was left from the patient’s perspective about the way to proceed. The narrative provided by the patient #P3F16 also illustrates how the caregiver focuses on the temporality of the treatment process. When caregivers stressed that the hospital stay would eventually come to an end, the adolescents reported to feel more confident in their abilities to undergo tedious treatment.

Apart from supporting adolescents emotionally, their caregivers can provide what can be called practical support at the point of admission to TB clinic. Such practical support can be expressed in packing a bag for a patient and subsequently bringing small gifts for cheering him or her up. Some caregivers indicated that they bought a new phone for their child to stay in touch. Furthermore, providing clarity on such practical matters as how a patient can contact a caregiver or when visits from caregivers can be expected also helps patients to feel more at ease upon arrival to the TB clinic.

Finally, caregivers can also provide cognitive support to adolescents at the beginning of their treatment. It refers to the patient’s need for disease- as well as treatment-specific information after having been diagnosed with TB. However, interviewed caregivers appear to be less well equipped to provide cognitive support, in comparison to the emotional and practical support, because of the caregivers’ own limits with regards to knowledge of TB. For example, one mother narrated:

Well, I was going with her [daughter] to the clinics, while she was doing the medical examinations. I helped her to get hospitalized. I was coming to her. What else can I do? I actually don’t understand anything about this. What else can I do? I was helping her, bringing her good food.

[#C4F]

This statement highlights that while most adolescents turn to their caregivers in order to comprehend what is going to happen to them, caregivers themselves often are empty-handed when it comes to fulfilling the cognitive needs of adolescent patients.

After the admission to the TB clinic, maintenance of regular communication with caregivers is paramount for the adolescents’ mental wellbeing. During the interviews adolescent patients emphasized the importance of their significant adults’ availability for contact in order to share concerns, worries, and good news with. It is this knowledge that there is someone who accepts them regardless of their TB diagnosis and is always there for them, that adolescents frequently fall back on during their treatment period. When speaking about their interactions, both adolescents and caregivers often mentioned open communication style. For example, one girl shared: ‘My mother understands me and tries to support me in everything. We discuss everything, so she knows everything about me’ [#P5F15]. Similar terms were used by her mother to describe her relationship with her daughter: ‘She tells me everything, she doesn’t have a secret from me. If she asks me, I will answer any of her questions.’ Many other patients similarly suggested that ability to discuss any topic and share any emotions with their caregivers is something that had helped them to adapt to staying in the TB clinic.

Adolescent patients and their caregivers use different modes of communication to interact with each other, including phone calls, text messages, and actual visits to the TB center. All patients interviewed indicated to have regular phone calls with their caregivers [at least once a week]. Typical is a two-way communication style, where the caregiver calls the patient, but also the patient initiates phone calls. The latter most often occurs when the adolescent received news, either negative or positive. For example, one patient shared her reaction to learning that she had to stay for three more months in the TB clinic: ‘Well, I called my grandmother’ [#P7F13]. The girl’s grandmother reassured her by promising to do her best to take her for home treatment. The girl shared with the interviewer that she felt calmed down after hearing the voice of her grandmother and knowing that she had her grandmother’s support. Adolescents’ desire to contact their caregivers the moment they are missing home or received news can be further illustrated by the narrative of a mother, where she described how she interacted with her hospitalized daughter:

We have a conversation every time she calls us. If something happens, she calls immediately, like ‘mom …’. She calls us with things like ‘I passed this; this happened …’, with every bit of news.

[#C5F]

When caregivers respond to the calls of patients, personal emotions can instantly be shared. Such sharing makes joyful emotions more joyful based on the positive reaction of the caregiver and helps to neutralize negative emotions by putting them into perspective and by reassuring the patient. Experiences shared by adolescent patients interviewed in this study highlight crucial importance of caregivers’ regular availability for contact when a patient needs to speak to them. But when such availability is lacking, hospitalized adolescents miss a central source of support for their mental wellbeing and struggle more.

While many caregivers answer every call and text of a hospitalized adolescent, initiate contact, and provide the patients with things requested, some may be less equipped to do so for a variety of reasons. An example of the latter is provided in the case of boy, who spoke to the interviewer on the day of his planned discharge from the TB clinic [#P1M14]. He arranged with his mother that he would be picked up from the clinic in order to return home. When his mother did not arrive at the pre-arranged time, the physician on duty decided to reach her over the phone and learned that the mother would not be arriving anymore that day, due to alcohol overconsumption. This case was exceptional. However, similar instances do happen and require serious attention because of their damaging impact on patients’ mental wellbeing.

Adolescents who enjoy responsive and stable relationship with their caregivers tend to interact with them more intensely at the beginning of the hospitalization. Over time, security provided by positive relationships with caregivers helps adolescents to adapt to the life in TB clinic and experience less need in constant interactions with caregivers. Whereas most adolescents claim they initially relied on their caregivers’ support to manage the anxiety of arriving at the TB clinic and continuously shared new things encountered in the unfamiliar environment, this emotional necessity diminishes after a couple of weeks. The patients’ narratives suggest that after some time they are getting used to their new surroundings and their daily routine, leaving them with little new information to share with their caregivers. Besides, a number of adolescents added that they began to spend more and more time with their peers at the clinic and developed new social connections. Illustrative here is a monologue of one of the caregivers who experienced a decreasing interest of her son to contact her as a result of him developing new relationships at the TB clinic:

We were talking every single day. By phone and I was coming very often. And in the end, I was coming just like, once in every two weeks. And then I was taking him home for the weekends. While we were talking daily, but the internet was not working that good, so we couldn’t talk by phone that much, but we were sending each other 300–400 messages a day. And then less and less. And then I was coming a bit later, I was just coming and giving him the gift and he was like ‘okay mom, goodbye’. They have friends there, they have parties. He doesn’t have time to talk to his mother.

[#C9F]

The timeline provided by this mother clearly shows how the caregiver’s initial interactions with her son shifted as the adolescent began to adapt to his immediate surroundings and build new friendships. The mother proceeded to describe how her worries about the condition of her son also subsided the moment she started to realize that he was making new friends and was feeling at ease about his stay at the TB clinic.

Overall, adolescent patients require most attention and support from their caregivers at the point of hospitalization and during the initial period of the stay at the TB clinic. They appreciate an opportunity to discuss any topic of importance for them and benefit much from regular availability of their caregivers for contact.

### Relationships with peers

Most interviewed adolescents had not heard much about TB prior to receiving this diagnosis. However, those few who had, became overwhelmed with anxious expectations of rejection by their friends and classmates once their diagnosis is known. This anxiety seems to be born out of attitudes towards TB and people with TB present in the communities where these adolescents grew up. For example, one of the adolescent patients learned about TB early in her life from her grandmother, who was a nurse in another TB clinic:

I used to sleepover at my grandmother’s place and she would tell me about tuberculosis, how scary this disease is. I therefore saw tuberculosis as something you cannot tell anyone about, because our people spread gossips all the time and quite fast. Half of my friends really like to gossip and if I tell them, everybody will know. I am afraid of being discussed by the people around me. I am afraid they will point their finger at me.

[#P5F15]

Upon learning about her own diagnosis this patient instantly decided to hide it, being unnerved by a prospect of becoming an outcast. She shared: ‘I simply kept my feelings for myself. I didn’t talk to anyone. I cried and stopped contacting my friends. Only one of my friends knows I have the disease and nobody else’.

While the decision to voluntary cut down social ties out of fear of rejection brought isolation and sadness to this girl, experiences of another adolescent patient who decided to disclose his diagnosis show that such fears are grounded. During the interview this patient described thinking upon learning that he had TB: ‘My friends will be very against me, they would refuse me. … My friends will start avoiding me, they will try to forget me’ [#P2M16]. Nonetheless, he decided to inform his friends about his health status. This patient reflected on the consequences of doing so with the following words:

Now I don’t have any contact with my friends at home who forgot me. In the past, I thought I had many friends. Now I only have one best friend from the past.

[#P2M16]

In both situations, where a patient decided to hide the TB diagnosis and where a patient decided to disclose, interpersonal relationships, central for mental wellbeing, suffered. As a result, these adolescents had to deal with the admission to the TB clinic without the support of their friends, feeling abandoned and even disdained. Negative views on TB, absorbed by these adolescents from their environments complicated acceptance of the diagnosis, made it harder to agree with the prospect of the long-term hospitalization, and often engendered negative feelings about oneself. Illustrative of a strain, experienced in such situations, is a statement of the girl who decided to hide she had TB [#P5F15]. She admitted feeling ‘hysterical’ by the time she arrived at the TB clinic because of loss of friendships and anticipation of further negative events caused by her contracting TB.

While not all interviewed adolescents experienced TB diagnosis in such devastating way, negative reactions from peers are not infrequent. Even patients who have not heard anything about TB prior to being evaluated for TB and did not originally expect rejection from their social circle, quickly pick up the view of this disease as something shameful and consequently, begin worrying. Importantly, this shaping of patients’ attitude towards their own diagnosis tends to happen before they meet their treating physician at the TB clinic but affects profoundly how patients initially experience hospitalization.

However, this unfavorable situation changes drastically once adolescents arrive to the TB clinic. The majority of the interviewed adolescents clearly remember the readiness with which they were approached by their peers already staying at the clinic. This active approaching stands out as vital for improving mental wellbeing of the newly admitted patients. One girl shared how unexpected but welcoming it was to be approached in a friendly manner by other patients directly upon entering the TB clinic ward:

Well you know, when I first came here, they examined me in the emergency room. And then they send me to the ward. I sat on the couch, my sister came to me. And I had a lot, a lot of girls around me and they started to ask me questions about my name, age, where I am from. I was kind of shocked, of course. To be honest, I thought I wouldn’t talk with anyone at all here.

[#P3F16]

Her story is echoed by the statement by another adolescent patient who described how such initial attention and openness by other patients helped her adapt to staying in the clinic. Whereas at first she did not want to stay in the TB clinic, the interactions with the other girls quickly made her feel better about the new environment:

I just came and they [the other girls] asked my name and how old I am, where I am from. Just all the girls here. … And then the next day I started talking with everyone. Everything was fine. Every day I was communicating more and more with everyone.

[#P7F13]

These narratives show how by actively seeking contact, already admitted adolescents provide support for the newcomers. It becomes easier for the latter to form new relations and interact with peers, which, in turn, takes the edge off their emotional state.

Indicative of the quality of new relations is the term ‘friends’ used by majority of the patients to refer to their peers in the TB clinic. When asked about positive aspects of their stay at the TB clinic, patients invariably mentioned acquiring new friends. This is also the case with the patient #P2M16 whose story of losing old friends after sharing his TB diagnosis was described above. This same patient continued his story:

During my time here, I realized who were really my friends and who were false friends. Here I have met some great people. I found some new friends.

[#P2M16]

While all adolescent patients look forward to getting cured and leaving the clinic, friendships developed in the course of the hospitalization can even make the discharge a bittersweet experience. One of the adolescents who somewhat regretted leaving the clinic is #P1M14. The interview with him took place on the day of his planned discharge. When asked to describe his feelings towards going home, he replied;

I feel a bit sad. I made friends in the clinic and I will miss them. But the kids here in the clinic are mostly from the city of Tomsk, so most likely we will meet each other again.

[#P1M14]

The strong and satisfying interpersonal relationships developed often persist beyond the TB clinic walls. A few weeks after the interview with #P1M14, an interview with his mother took place. During this interview the mother told how her son was still visiting the TB clinic on a regular basis. Also, that day, #P1M14 joined his mother to the clinic to meet up with the friends he had made during his stay there. In the words of the mother about her son:

He likes this place. … New people around, he likes it. Of course, it is a hospital, but I can see that they have a lot of fun here. And he still goes here to visit his friends.

[#C1F]

What makes the relationships between adolescents who receive treatment at the TB clinic so positive is the non-judgmental attitude towards each other that seems to be prominent. They all share similar health condition which differentiates them from friends back home, contacts with whom are often broken after TB diagnosis is pronounced. Relations among adolescent patients in the TB clinic are marked with what is often missing in relationships with peers outside the clinic–acceptance. One adolescent patient reflected on the quality of relations she developed in the TB clinic:

Good communication, good relationships with the other girls. I like the contact with the smaller children. We don’t argue. We understand each other well. We don’t gossip about anyone.

[#P5F15]

In the clinic, having TB does not threaten one’s social standing and does not cause rejection because there everyone is being treated for TB; hospitalized adolescents don’t judge each other for having this diagnosis. This accepting attitude is paramount for patients’ psychological wellbeing as it promotes a positive image of self, which could be damaged by pre-hospitalization experiences of being rejected by peers.

The positive impact of having peers around from moment one onwards can be seen even more clearly when comparing mood and functioning of adolescents who reside in the general ward with those of adolescents who start their therapy in an isolation ward. The isolation ward hosts patients with contagious drug resistant forms of TB. Once contagion is not a concern anymore, these patients move to the general ward. Patients who underwent treatment in isolation describe their initial period at the TB clinic as an extremely difficult one. For example, both #P8F15 and #P6M15 were first treated in isolation. They reflect upon their period in the isolation ward by saying:

It was hard for me to be in an unknown place, absolutely alone. In a closed environment. That is it. … I was crying a lot, was being sad. I felt disappointed.

[#P8F15]

It is so hard to stay in the isolated ward for five months. To not talk with anyone, to not go anywhere. No friends, no relatives. I had changes of mood pretty often.

[#P6M15]

Loneliness and sadness were emotions adolescents regularly referred to when speaking about their time in the isolation ward, whereas adolescents in the general ward described having much more positive affect and more satisfaction with their daily life. In the isolation, patients typically feel disappointed and frustrated with themselves and their health condition, in contrast to general ward where the prevalent non-judgmental attitude facilitates acceptance of diagnosis and treatment process as well as more positive image of self. Adolescent patients describe quickly starting to feel better, once transferred from the isolation to the general ward. Still, time in the isolation, away from peers, carries profound negative impact on the adolescents’ mental wellbeing.

One thing that adolescent informants did not mention during interviews was arguments and conflicts among patients. None of the adolescents referred to a situation in which others or they themselves were involved in a quarrel. All physicians however mentioned the regularly occurring personal conflicts between the children. Being aware of this possibility, physicians reported actively looking out for signs of confrontation to tackle it at birth. Illustrative here is an example provided by one physician, who described how she acts the moment she notices a conflict emerging between patients:

If I see a very tense atmosphere amongst some children, I am trying to gather them all, for example all three members of this conflict. And we all come together and discuss this problem all together. We are organizing a meeting to resolve the conflict in an open manner. Because it is really important to look into their eyes, because it is really complicated to find out who is really guilty, that is why an open discussion is the only solution.

[#D2F]

Given that adolescents stay at the TB clinic for a prolonged period of time, being surrounded by a relatively constant group of people, it is likely that quarrels do occur even though no adolescent patients mentioned them. Physicians take it upon themselves to monitor and manage conflicts between patients, considering, as was already mentioned, positive mood and supportive atmosphere important for patients’ recovery.

Overall, adolescent patients benefit enormously from peer support, typical absence of which in their usual social environment is highly traumatic. Distressed newcomers feel relieved when other adolescents seek their company and appreciate it when their health condition is taken neutrally, as something that does not ultimately define the character of their relationships with others.

## Discussion and conclusion

The results of our study highlight that threats to mental wellbeing of adolescents with TB are multiple. Some of them, including drastic change of routines and environment following treatment initiation, are directly related to hospitalization. Others, such as lack of social acceptance, are tied to the TB diagnosis itself but influence the hospitalization experience as well, at least initially. Relationships with others, though, have a potential to support mental wellbeing of hospitalized adolescents in all three core domains—emotional, psychological, and social [40].

Predominant emotional state among adolescents who learned about their diagnosis and are about to enter in-patient treatment in the TB clinic is characterized by anxiety and sadness. They feel apprehensive towards their future, unsure about what it will bring and fearing they would not be content with their life. In terms of psychological functioning, adolescents tend to have a hard time accepting their diagnosis, which they often feel is something shameful, and, consequently, may develop a negative attitude towards themselves. Previous studies highlighted similar risk of internalizing TB-associated stigma pertinent specifically to adolescents [15]. This study, additionally, showed how tuberculosis and its treatment were experienced as a threat to their direction in life and exacerbated the feeling of helplessness brought in by decreased degree of self-determination associated with in-patient treatment. Perhaps most importantly, many adolescents undergo painful loss of personal relationships and expect or actually experience rejection by peers because of having TB. Challenges in emotional and psychological domains are intimately tied to social functioning, which suffers from disarray in social life and loss of sense of belonging.

Our results show that relationships with physicians, caregivers, and peers mediate negative impacts of TB diagnosis and hospitalization on adolescents’ mental wellbeing and can open ways for providing support. Previous research pointed to hostility and anger as typical reactions of adolescents to hospitalization [4, 5]. Lack of these reactions observed in this study may, at least partly, be explained by the presence of supportive relationships. When physicians leave it up to adolescents to decide when they like to speak to physicians, which topics they like to discuss and how emotionally open they like to be, adolescents gain enhanced sense of autonomy and control over at least some aspects of their daily life, which is an important component of psychological wellbeing [17]. Psychological wellbeing of at least some adolescent patients appears to be further enhanced by immediate availability of physicians to answer their questions. Through acquiring up-to-date information about their health state and treatment progress adolescents feel more confident and competent with respect to one of the most valued life goals at the moment—getting healthy. Regular availability of caregivers for contact is of tremendous importance for psychological wellbeing of hospitalized adolescents, since stable and satisfying relationships are at the core of positive psychological functioning. When caregivers reaffirm their commitment to patients through availability, providing practical support, and initiating and responding to phone communications, adolescents are reassured of the strength and warmth of their relationship and feel calmer. Finally, friendliness and non-judgmental attitude of other adolescents with TB helps patients to feel better about themselves and feel part of a community. Comfort derived from a sense of belonging is a powerful facilitator of positive social functioning [30]. All these practices by physicians, caregivers, and peers also contribute to emotional wellbeing of hospitalized adolescents, who report feeling happier and more content as a result.

Research done in the recent years affirms that not all children and adolescents diagnosed with TB need to be hospitalized for treatment [41, 42]. However, opinions diverge as to in which situations hospitalization is desirable. What is clear is that to decide on the treatment modality in practice, physicians have to weigh a number of considerations that extend beyond purely medical ones. And sometimes due to either the severity of medical condition, or lack of adequate care at home or community, or some combination of both, the scales may point towards hospitalization. In such situations in-patient care can be made more adolescent-friendly by, firstly, designing explicit measures to support caregiver-patient relationships through, for instance, reimbursing travel costs for those who live far away, informing caregivers about ways to support mental wellbeing of hospitalized adolescents, and creating activities that encourage interactions between caregivers and patients such as dinners and movie nights where both parties can be present. Regular counselling for both patients and caregivers can be necessary. Secondly, in-patient TB treatment can be tailored to the needs of adolescents by creating more decision-making opportunities for the adolescents. Such opportunities can include choice of times, topics, and formats for interactions with physicians, as is done in Tomsk pediatric TB clinic, but can also go beyond to include choices in daily schedule and extracurricular activities. TB treatment routines can further incorporate regular screening for mental health concerns, given the significant stressors of prolonged hospitalization and separation from parents, particularly for the adolescents who are in isolation. Thirdly, importance of adolescent patients’ relationships with peers needs to be recognized for making care more adolescent friendly. While it is difficult to institute measures that would target relationships between adolescents, options can include systematic creation of culture of acceptance through conversations and own behavior by clinic staff, design of the clinic with multiple common areas, and provision of support to group activities and interest clubs initiated by the patients themselves. A particularly acute challenge is social life of the adolescents in the isolation ward. Apart from reconsidering the length of isolation, it is essential to find ways to allow adolescents in the isolation ward to interact with other patients. One way of doing so is with the use of digital technologies; there can be regular online group socializing sessions organized with other adolescents in the hospital, both in isolation and general wards. These sessions would require distribution of tablets and/or installation of computers with large screens in accessible areas and can be devoted to introductions, when a new patient arrives, games, and support meetings.

However, threats to mental wellbeing of adolescents with TB come not just from the hospitalization itself. We demonstrated how rejection by peers that precedes hospitalization poses a very real threat. Popular negative perceptions of TB lead adolescents with TB to doubt their self-worth and wreak havoc in their friendships. Paradoxically, hospitalization can provide an opportunity to find supportive and accepting peers, thus mitigating some most important threats to mental wellbeing. At the same time, the practice of long-term hospitalization itself can be contributing to the stigmatization of people with TB, indicating that they are not to stay in society. Therefore, apart from instituting more diverse and responsive treatment trajectories for adolescents with TB so that alternatives to hospitalization can be considered, it is imperative to develop suitable interventions to support mental wellbeing of those adolescents, whose treatment outside the hospital may prevent them from finding a new community of understanding peers. Such interventions can include peer support groups and regular counselling attentive to the issues of social connectedness.

### Limitations

This study has several potential limitations. Sampling strategy was guided by participants’ availability and was restricted to 17 informants including 8 adolescent patients. Therefore, the obtained results provide an in-depth understanding of a range of mental health challenges which hospitalized adolescents with TB experience and of a range of support strategies which physicians and caregivers employ. The results are not informative with regards to prevalence of certain views and behaviors. The involvement of an interpreter may have resulted in information omissions. At the same time, interpreter trained in qualitative interviewing and closely familiar with local healthcare environment helped the interviewer to understand local context, which is important for ensuring quality of data collection. Additionally, the study would have benefitted from formal interviewing of other hospital staff such as nurses, psychologists, and teachers. In this study they were involved only through informal non-structured conversations held in the process of fieldwork.

## Supporting information

S1 File Interview guide for patients. (DOCX)
