# Supplementary material for: Adolescents in a tuberculosis hospital: Qualitative study of how relationships with doctors, caregivers, and peers mediate their mental wellbeing
Source: PLoS One. 2021 Oct 1;16(10):e0257379. doi: 10.1371/journal.pone.0257379 (PMC8486124; doi:10.1371/journal.pone.0257379)
Supplement: S1 File — (DOCX) [file pone.0257379.s001.docx]

## Interview guide for patients/Путеводитель для интервью с пациентами

## Introductory questions/Вводные вопросы

1. How did you become ill?/Расскажи, как ты заболела?

- PROBE – How did you realize something might be wrong with your health?/Как ты поняла, что, возможно, заболела?
- PROBE – When did you go to a doctor?/ Когда ты пошла к врачу?
- PROBE – What happened when you went to a doctor?/ Что произошло, когда ты пришла к врачу?

1. How did you find out you would have to be admitted to the medical center?/ Как ты узнала, что тебе предстоит отправиться в больницу?

- PROBE – How did it make you feel? /Что ты почувствовала?
- PROBE – What were your thoughts before coming to the medical center? /О чем ты думала перед тем, как приехать сюда (в больницу)?
- PROBE – What did you worry about? / Тебя что-либо волновало? Что?
- PROBE – What did you find the most difficult? /Что было для тебя тяжелее всего?

## Main questions/Основные вопросы

1. How would you describe your parents? [*characteristics]*  Расскажи о своих родителях

- PROBE – How did you get along with your parents prior to falling ill?/ Как вы общались с родителями до того, как ты заболела?
- PROBE – What do you like in your parents behavior? /Что тебе нравится в твоих родителях?
- PROBE – What don’t you like in your parents behavior?/ А что не очень нравится?

1. How did your parents hear about your diagnosis? / Как твои родители узнали о твоем диагнозе?

- PROBE – How did your parents respond to the diagnosis?/ Как они отреагировали?
- PROBE – How did your parents reaction make you feel? / Что ты почувствовала, когда они .. (реакция родителей)?
- PROBE – Do your parents treat you different after you fell ill? If so, how? / А твои отношения с родителями изменились после того, как ты заболела, или нет?

1. What happened after you got diagnosed with tuberculosis? /Что произошло после того, как тебе поставили диагноз «туберкулез»?

- PROBE – How did your life change? /Изменилась ли твоя жизнь? Как?
- PROBE – How did your parents help you? [*period between diagnosis and admission at medical center]* / Как родители помогали тебе?
- PROBE – How did it make you feel? / Что ты чувствовала при этом?

1. How is it to stay here in the tuberculosis medical center? / Как тебе здесь, в больнице?

- PROBE – How does it make you feel? / Как ты себя здесь чувствуешь?
- PROBE – What does make you feel that way? / Почему?
- PROBE – Did your feelings change compared to your first days here? If so, how? /А в самом начале, в первые дни, как ты себя чувствовала?
- PROBE – What do you like about your stay here? /Что тебе здесь нравится?
- PROBE – What do you find difficult about your stay here? /А что было сложным для тебя здесь?

1. How are doctors here? / Что ты думаешь про врачей здесь?

- PROBE – What did your first encounter with a doctor look like? /Расскажи про свою первую встречу с врачами?
- PROBE – How did it make you feel? / Что ты почувствовала?
- PROBE – Did your feelings change compared to the first day here? If so, how? /А сейчас ты чувствуешь себя так же или по-другому?

1. What happens when you see a doctor now? /Что происходит, когда ты встречаешься с врачом?

- PROBE – What do you discuss with your doctor? /О чем вы разговариваете?
- PROBE – Are there topics you cannot discuss with your doctor? If so, what topics? /А есть ли темы, которые ты не можешь обсуждать со своим доктором? Какие?
- PROBE – What do you like about your contact with your doctor? /Что тебе нравится в твоих отношениях со своим врачом?
- PROBE – What don’t you like about your contact with your doctor? /А что не нравится?
- PROBE – What would you like to change in your contact with your doctor? /Тебе хотелось бы что-то изменить в своих отношениях с врачом?

1. How do you have contact with your parents now? /Как ты общаешься со своими родителями сейчас?

- PROBE – What kind of topics do you discuss with your parents? /О чем вы разговариваете?
- PROBE – What do you like in your communication with your parents? / Что тебе нравится в вашем общении?
- PROBE – What do you not like in your communication with your parents? /А есть ли что-то, что не нравится?
- PROBE – What happens when your parents are here? /Что происходит, когда твои родители приезжают сюда?
- PROBE – How does it make you feel? / Что ты чувствуешь?
- PROBE – Did the communication between you and your parents change during your stay here? If so, how? /Изменилось ли ваше общение с родителями за то время, пока ты здесь? Или нет? Как?
- PROBE – Are there topics you would like to discuss with your parents, but that you cannot? If so, what topics? / А есть ли темы, которые тебе хотелось бы обсудить со своими родителями, но ты не можешь?

## Closing questions/Завершающие вопросы

1. Is there anything else that you would like to tell me? /Есть ли что-то еще, что ты хотела бы мне рассказать?
2. Do you have any remaining questions for me? / У тебя есть ко мне вопросы?
